# A Bayesian approach to estimating COVID-19 incidence and infection fatality rates

**DOI:** 10.1093/biostatistics/kxad003

**Published:** 2023-03-06

**Authors:** Justin J Slater, Aiyush Bansal, Harlan Campbell, Jeffrey S Rosenthal, Paul Gustafson, Patrick E Brown

**Affiliations:** Department of Statistical Sciences, University of Toronto, 700 University Avenue, 9th Floor Toronto, ON M5G 1Z5, Canada; Centre for Global Health Research, St. Michael’s Hospital, 30 Bond Street, Toronto, ON M5B 1W8, Canada; Department of Statistics, University of British Columbia, 2207 Main Mall, Vancouver, BC V6T 1Z4, Canada; Department of Statistical Sciences, University of Toronto, 700 University Avenue, 9th Floor Toronto, ON M5G 1Z5, Canada; Department of Statistics, University of British Columbia, 2207 Main Mall, Vancouver, BC V6T 1Z4, Canada; Centre for Global Health Research, St. Michael’s Hospital, 30 Bond Street, Toronto, ON M5B 1W8, Canada and Department of Statistical Sciences, University of Toronto, 700 University Avenue, 9th Floor Toronto, ON M5G 1Z5, Canada

**Keywords:** Bayesian analysis, COVID-19, Modular inference, Mixture model, Post-stratification

## Abstract

Naive estimates of incidence and infection fatality rates (IFR) of coronavirus disease 2019 suffer from a variety of biases, many of which relate to preferential testing. This has motivated epidemiologists from around the globe to conduct serosurveys that measure the immunity of individuals by testing for the presence of SARS-CoV-2 antibodies in the blood. These quantitative measures (titer values) are then used as a proxy for previous or current infection. However, statistical methods that use this data to its full potential have yet to be developed. Previous researchers have discretized these continuous values, discarding potentially useful information. In this article, we demonstrate how multivariate mixture models can be used in combination with post-stratification to estimate cumulative incidence and IFR in an approximate Bayesian framework without discretization. In doing so, we account for uncertainty from both the estimated number of infections and incomplete deaths data to provide estimates of IFR. This method is demonstrated using data from the Action to Beat Coronavirus erosurvey in Canada.

## 1. Introduction

As of April 1, 2022, there have been close to 500 million confirmed cases of coronavirus disease 2019 (COVID-19) worldwide (World Health Organization, 2022). However, the general consensus is that this number is an underestimate of the true cumulative incidence of the disease, as this estimate is largely dependent on the number of tests being administered, the accuracy of testing (Burstyn *and others*, 2020a,b), and to whom these tests are being issued. If testing is extensive enough, and a correction is made for underreporting of asymptomatic cases, then a test-based case fatality rate may be a reasonable proxy for the infection fatality rate (IFR) (Luo *and others*, 2021). However, given that the testing early in the pandemic was sparse, and estimating IFR accurately is of the utmost importance, epidemiologists across the globe are conducting serosurveys that measure immunity of individuals by testing for the presence of SARS-CoV-2 antibodies in the blood (Chen *and others*, 2021). This quantitative measure (which we will call a *titer value*) is then used as a proxy for previous or current infection. However, how exactly these data should be used to accurately estimate important epidemiological quantities (like incidence and IFR) is an active area of research.

The standard approach is to label everyone who has a titer value above some threshold as “infected” and consider everyone else not infected. This leads to the problem of selecting the cutoff, which can be made based on known cases/controls and analysis of the receiver operating characteristic (ROC) curve. The ROC plots the true positive rate (sensitivity) versus the false positive rate (1-specificity), and it is typical to select the cutoff that results in the highest Youden Index (sensitivity + specificity $-$ 1) (Krzanowski and Hand, 2009). Gelman and Carpenter (2020) suggest that the uncertainty in sensitivity and specificity can be considered parameters to be estimated in a Bayesian hierarchical model assuming that informative priors are used for the sensitivity and specificity. Although this method accounts for uncertainty in the sensitivity and specificity, it still suffers from the loss of information in the discretization process. Particularly in COVID-19 applications, a subject with an extremely high level of antibodies should have a lower probability of being a false-positive than someone who is just barely above the threshold. This could be partially remedied by allowing sensitivity and specificity to be a function of covariates, but ideally methods that avoid these issues all together are preferable.

Mixture models are a natural choice to overcome the limitations of using a fixed cutoff, as they allow infection status and associated uncertainty to depend on the magnitude of individuals’ titer values. Mixture models have been widely applied when studying the prevalence of infectious diseases in animals (Ødegård *and others*, 2003; 2005; Nielsen *and others*, 2007) and in humans (Vink *and others*, 2015, 2016; Kyomuhangi and Giorgi, 2022). There are several other papers that have modeled the COVID-19 antibody levels directly to infer cumulative incidence through the use of mixture models. Bouman *and others* (2021) showed that mixture models can outperform the methods of Gelman and Carpenter (2020) for estimation of cumulative incidence of COVID-19. Furthermore, Bottomley *and others* (2021) apply mixture models to Kenyan serosurvey data and show that mixture of skew normal distributions more accurately estimates cumulative incidence than methods based on thresholds. However, the applications of these models thus far has been rather limited. For instance, some unexplored questions include: how do we use these mixture models to account for survey bias and get cumulative incidence rates for the general population? How do we incorporate multiple titer values per person? How do we estimate cumulative incidence in the presence of vaccinated individuals? How do we use these mixture models to estimate IFR while accounting for uncertainty in both the number of infections and deaths?

In this article, we demonstrate how mixture models can be used to estimate cumulative incidence in an approximate Bayesian framework without discretization. Specifically, we apply a mixture of multivariate t-distributions to the log of the titer values, using a logistic regression model for the mixing parameter to account for covariates. We then use post-stratification to obtain estimates of cumulative incidence and its associated uncertainty. Furthermore, we estimate the number of COVID-19-related deaths using partially complete data and use this in combination with incidence estimates to estimate the IFR across Canada.

### 1.1 Data

Dry blood spot (DBS) samples were collected from participants of the Action to Beat Coronavirus (Ab-C) study (https://www.abcstudy.ca/). This article is concerned with the first two *phases* of the study. In Phase 1, DBS samples from 9123 participants were collected from June to November 2020 and roughly corresponding to the first viral wave (April 1–July 31, 2020). In Phase 2, DBS samples from 7299 were collected from December 2020 to May 2021 and roughly correspond to the second viral wave (October 1, 2020–March 1, 2021). These blood spots were tested for prevalence of immunoglobin G (IgG) antibodies, measured using three antigens: Spike (SmT1), RBD, and nucleocapsid (NP). Two different versions of the SmT1 antigen test were used on the Phase 1 blood spots, while all three were applied to Phase 2 blood spots. All three titers will show larger values for participants who have been exposed to COVID-19, but only SmT1 and RBD will show larger values for mRNA vaccinated individuals. This is because the mRNA vaccines do not contain the nucleocapsid (NP) protein. Therefore, people who received an mRNA vaccine and did not have a history of prior infection, will not develop anti-NP antibodies. Those that were previously infected, regardless of vaccination status, will have anti-NP antibodies (Houlihan and Beale, 2020). This will be helpful for distinguishing between vaccinated and infected individuals in Section 3.3. In Phase 1, 8919 people had one SmT1 measurement, and 8704 had two SmT1 titer measurements, along with complete covariate information. In Phase 2, 7065 had all three measurements, along with complete covariate information. Of those 7065, 624 joined the study in Phase 2 (6441 participants had complete Phase 1 and Phase 2 data). These data have been previously analyzed by Tang *and others* (2022) using a simpler model. Additional medical details regarding these antigen tests can be found in their paper. Tang *and others* (2022) also investigated the representativeness of study participants when compared to the Canadian population. They found that the study population tended to be older, more university educated, more likely to be indigenous, etc. See eTable 3 in their paper for further reading.

Although serosurveys are a proven way to accurately measure seroprevalence, the notion of seroprevalence itself has several drawbacks. Firstly, there is a chance that participants got infected and returned their blood spots soon after. Antibodies generally take between 7 and 14 days to be measurable from the onset of infection (Centre for Disease Control and Prevention, 2022). This may cause a slight under-estimation of incidence. Secondly, antibodies wane slowly over time. However, they have been shown to remain elevated for many months after infection. In a study (Alfego *and others*, 2021) evaluating 39 086 individuals with confirmed positive COVID-19 infection by RT-PCR between March 2020 and January 2021, the anti-NP antibody remained elevated in 68.2$\%$ [95$\%$ Cl: 63.1–70.8] of participants after 293 days, while anti-SmT1 antibody remained elevated in 87.8$\%$ [95$\%$ Cl: 86.3–89.1] of participants after 300 days. Note that the majority of people in our study were likely infected far less than 300 days prior to submitting their blood spots, so the maintenance percentage in our study was likely higher than those in Alfego *and others* (2021). At this point, we simply note these limitations of seroprevalence, and examine the potential impact of waning immunity on our results in Appendix F.

Population demographics (age, sex, province, ethnicity, education, and long-term care residency) were obtained from 2016 Census data from Statistics Canada (Statistics Canada, 2016). We are using the 2016 Census data because, at the time of writing, the 2021 Census data pertaining to education and ethnicity was not available. The age/sex/geographic data for 2021 were available and while the total population increased roughly 5$\%$ between 2016 and 2021, the age-sex and geographic distributions were nearly identical. This information will be used for post-stratification as described in Section 2.3. The long-term care (LTC) COVID-19 deaths were obtained from https://ltc-covid19-tracker.ca (Samir *and others*, 2022) between September 2020 and March 2021 for each province. The total deaths for each province by age and sex were obtained from the different provincial governments (Ontario, Alberta, and Quebec). For additional provinces, where deaths by age and sex could not be obtained, we used the distribution of nearby provinces to approximate those deaths. The age/sex distribution of deaths in Alberta was used to infer the distribution of deaths in British Columbia and Saskatchewan. The age/sex distribution of deaths in Quebec was used to infer the distribution for the Atlantic region (New Brunswick, Nova Scotia, Newfoundland, and Prince Edward Island). Manitoba reported different age groups than Ontario but seemed to have a similar distribution. Thus, we used Ontario data to infer Manitoba’s age/sex deaths for the different age groups. This means that although the aggregate IFR estimates for the Atlantic region, Manitoba, British Columbia, and Saskatchewan are likely valid, the estimates by age/sex should be treated with caution due to the imputations noted above.

## 2. Methods

Our first goal is to estimate the cumulative incidence of SARS-CoV-2 in Canada. We define *cumulative incidence* in Phase 1 to be the number of SARS-CoV-2 infections up until September 30th 2020, divided by the population size. The cumulative incidence in Phase 1 and 2 has the cumulative number of infections up until March 31st 2021 as the numerator. We define the *incidence proportion* in Phase 2 to be the number of infections from October 1st 2020 to March 31st 2021, divided by the population size. We recognize that the terms cumulative incidence and incidence proportion are used interchangeably in the epidemiology literature, and we are avoiding the term “cumulative” when presenting estimates of incidence in Phase 2 alone. We estimate incidence in two steps. First, we will fit a Bayesian mixture model to the titer values, relating an individual’s infection status, a latent variable, to their measured covariates via a logistic regression model. Second, we will use post-stratification to account for the disparity between the population of survey responders versus the general Canadian population. This will yield an estimate of the number of infections in Canada for each covariate combination, and hence, an estimate of the cumulative incidence.

Our second goal is to estimate the *Infection Fatality Rate*, which is defined as the number of COVID-19 related deaths divided by the number of infections. This will be estimated in Phase 1, Phases 1 and 2, and Phase 2 alone with the same time periods as mentioned previously. We do this by building a Bayesian model for the number of deaths in Canada by age/sex/province group, and dividing this by the estimated number of infections. This will allow for estimates of IFR in any age/sex/province category that we want, accounting for uncertainty in both the deaths and the infections.

### 2.1 Notation

Lower case Latin letters are used to represent (potentially vector-valued) observed data; $x$ are observed covariates, $w$ is observed titer values, and $d$ is observed deaths. The exception is $p$, which is an unknown probability of infection. Upper-case Latin letters represent latent variables (“missing data”), such as the unknown number of infections $Y$, an unknown number of deaths $D$, and the latent infection status $Z$ of an individual. Greek letters will be used for model parameters.

### 2.2 Mixture models

In this subsection we will introduce three mixture models that will be used to infer cumulative incidence. First, we will introduce a univariate (one titer value), two-component (“not infected” and “infected”) mixture model, relating each study participant’s covariates to their probability of infection. We will then extend this model to the bivariate case with two titer values in Section 2.2.2. These two models will be fit to the Phase 1 data. We will then present a trivariate, three-component (“unvaccinated, not infected,” “unvaccinated, infected,” and “vaccinated, not infected”) mixture model that will be fit to the Phase 2 data. Note that the “infected” group here contains both vaccinated and unvaccinated people as our titers values are not precise enough to determine vaccination status if a person is infected. This is likely inconsequential as we will explain shortly.

#### 2.2.1 Univariate mixture of t-distributions—Phase 1.

**Fig. 1.**
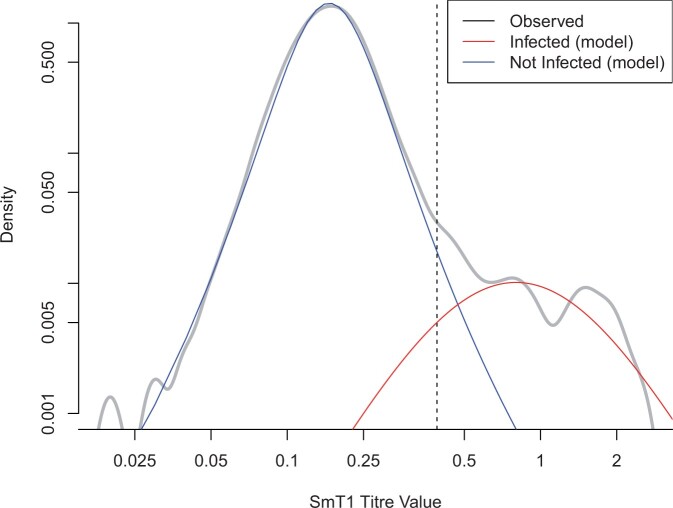
Mixture of t-distributions for the Phase 1 univariate model fit to the SmT1 titer values. The posterior median for each parameter is used. The vertical dashed line represents the cutoff used in Tang *and others* (2022). Keep in mind that this plot does not display uncertainty in the model parameters of the t-distributions.

The infectivity status, $Z_i$, of an individual $i$ is latent and is measured through an antibody lab test (titer), which is a quantitative measure. The density of the logged Phase 1 SmT1 titer values is shown in Figure 1. Notice that there is an approximately symmetric mound around $0.15$ which is likely to be comprised of individuals who never had COVID-19. Previously, Gaussian distributions were used to model the logged titer values in non-infected individuals (Bottomley *and others*, 2021). However, we expected a heavier-tailed distribution would be needed, and employ a t-distribution for both the negative and positive individuals.

The univariate, two-component version of our mixture model can be written as follows:


(2.1) \begin{align*}
\log(w_i)|Z_i=k &\sim f_1(\mu_k, \sigma_k, \nu_k), k=0,1 \nonumber\\
Z_i|x_i &\sim \text{Bernoulli}(p_i)\\
\text{logit}(p_i) &= \beta^Tx_i \nonumber
\end{align*}

where $w_i$ is the titer value of individual $i$, $Z_i$ is the latent variable indicating SARS-CoV-2 infection ($Z_i=1$) or non-incidence ($Z_i=0$), $x_i$ is a $m \times 1$ vector of covariates, $\beta$ is a $1 \times (m+1)$ vector of regression coefficients which will be used for post-stratification as described in Section 2.3, $f_1$ is the univariate (shifted and scaled) t-density, and $p_i=\text{logit}^{-1}(\beta^T x_i)$ is the probability that individual $i$ has been infected with COVID-19. That is, the probability that someone had COVID-19 is a function of their covariates, but the parameters of the t-distributions are not. The covariates used in our mixture models were age ($<$20, 20–29, 30–39, 40–49, 50–59, 60–69, 70–79, and 80+), sex (male, female), province (Alberta, Atlantic Region, British Columbia, Manitoba, Ontario, Quebec, and Saskatchewan), ethnicity (white, indigenous, not white or indigenous), and education (university degree, college degree, and less than college degree), meaning that $m=18$.

Since $Z_i$ is a latent discrete variable, certain MCMC software programs cannot sample it directly. However, we can marginalize $Z_i$ out to obtain the following likelihood:


$$
\pi(\log(w_i);\beta ,\boldsymbol{\xi}, x_i)= [1-\text{logit}^{-1}(\beta^Tx_i)] f_1[\log(w_i)|\mu_0, \sigma_0, \nu_0] + \text{logit}^{-1}(\beta^Tx_i) f_1[\log(w_i)|\mu_1, \sigma_1, \nu_1],
$$

where $\boldsymbol{\xi} = \{\mu_0,\mu_1, \sigma_0,\sigma_1,\nu_0,\nu_1\}$ is a vector of parameters which need to be estimated but are not used to infer incidence directly.

For both Phase 1 and Phase 2, we have continuous values for multiple titers and thus will now extend this univariate mixture model to a mixture of multivariate t-distributions.

#### 2.2.2 A bivariate mixture model for Phase 1.

For Phase 1, we have two measurements of SmT1 for each sample. Using both titers should improve our ability to identify individuals who were infected. Our model naturally extends to the bivariate case by replacing the univariate t-distribution by a bivariate t-distribution ($f_2$):


(2.2) \begin{align*}
\label{eq:MVT}
\log(\boldsymbol{w}_i)|Z_i=k, x_i &\sim f_2(\boldsymbol{\mu}_k,\boldsymbol{\Sigma}_k, \nu_k), k=0,1 \nonumber\\
Z_i|x_i &\sim \text{Bernoulli}(p_i)\\
\text{logit}(p_i) &= \beta^Tx_i \nonumber
\end{align*}

where $\boldsymbol{\mu}_k$ is a vector of length 2, $\Sigma_k$ is a $2\times2$ covariance matrix, and the rest of the parameters are the same as Section 2.2.1. Note that the logistic regression model for $Z_i$ in the second level is still univariate. This allows the model to accommodate multiple titer values per person without the number of parameters getting out of control. We fit this bivariate model on the two Phase 1 titer values using MCMC to obtain posterior samples of $\beta$ which will be used later for post-stratification.

#### 2.2.3 A trivariate, three-component mixture model for Phase 2.

In Phase 1, vaccinations had not yet been made available and $Z_i$ could only take on two values: “infected” or “not infected”. However, during Phase 2, a non-negligible proportion ($\approx$ 2.5$\%$) had claimed to have been vaccinated. Given that vaccinated people are distinguishable from infected people based on the three titer values that we have available, we now have three mutually exclusive values for $Z_i$: “unvaccinated, not infected,” “unvaccinated, infected,” and “vaccinated, not infected.” We did not include a fourth group “vaccinated, infected,” as there were likely to be very few participants in this category. Note that we can differentiate between “vaccinated, not infected” and “unvaccinated, infected” individuals because infected individuals will tend to have high titer values for all three titers, while vaccinated individuals should not have an elevated titer value for NP. That is, if a participant shows a high value of SmT1 and RBD, and a low value for NP, it should predict a small probability of infection. If a participant has a large value for all three, then the model should predict a large probability of infection.

Furthermore, we decided not to use self-reported vaccination status as data, as only about half of the participants who claimed to be vaccinated were showing large values of SmT1 and RBD. This may be because they had only received one dose, or perhaps they had provided their blood spot less than 2 weeks since their second dose. Either way, we want the data (titer values) to determine SARS-CoV-2 incidence, rather than rely on self-reported claims of vaccination.

In addition to having three infected statuses, we also now have three titer values which we can use to define a mixture of three trivariate t-distributions ($f_3$). The likelihood for this trivariate model is:


\begin{align*}
\pi(\log(\boldsymbol{w}_i);\beta ,\boldsymbol{\xi}, x_i) & = (1-\rho)[1-\text{logit}^{-1}(\beta^Tx_i)] f_3(\log(\boldsymbol{w}_i)|\boldsymbol{\mu}_0, \boldsymbol{\Sigma}_0, \nu_0) \\
&\quad{} + \text{logit}^{-1}(\beta^Tx_i) f_3(\log(\boldsymbol{w}_i)|\boldsymbol{\mu}_1, \boldsymbol{\Sigma}_1, \nu_1) \\
&\quad{} + \rho[1-\text{logit}^{-1}(\beta^Tx_i)] f_3(\log(\boldsymbol{w}_i)|\boldsymbol{\mu}_2, \boldsymbol{\Sigma}_2, \nu_2),
\end{align*}

where $\rho = \text{Prob}(y_i=2|y_i\neq 1)$. Here, $\text{Prob}(y_i=0) = \text{Prob}(y_i=0|y_i \neq 1) \text{Prob}(y_i \neq 1)= (1-\rho) (1-\text{logit}^{-1} (\beta^T x_i))$. We fit this trivariate model to Phase 2 data using Bayesian MCMC to obtain posterior samples of $\beta$ which will be used for post-stratification.

### 2.3 Estimating incidence using post-stratification

Incidence is defined as the number of people with an infection in a given time frame, divided by the population. We estimate incidence of COVID-19 in a subgroup of Canadians $G$ by taking posterior samples of $I_G$ where


$$
I_G = \frac{\sum_{h\ell j\in G} Y_{h\ell j}}{\sum_{h\ell j\in G} n_{h\ell j}} = \frac{Y_G}{n_G},$$

$h$ is ethnicity/education, $\ell$ is age/sex, $j$ is province, $p_{h \ell j}$ is the probability of COVID-19 infection (as in Equation 2.2) for a person with covariate combination $h \ell j$, $Y_{h\ell j}$ is the number of people in Canada with covariate combination $h \ell j$ who were infected with COVID-19, and $n_{h\ell j}$ is the number of people in Canada with covariate combination $h \ell j$. To obtain samples of $I_G$ we first fit the mixture models presented in Section 2.2 to obtain $T$ posterior samples of $p_{h \ell j}$. We then use post-stratification (Little, 1993) to generalize these results to the Canadian population. That is, we draw one sample from


$$
Y^{(t)}_{h \ell j} \sim \text{Bin}(n_{h \ell j}, p^{(t)}_{h \ell j})
$$

for each $t=1...T$. We then compute


$$
I^{(t)}_G = \frac{\sum_{h\ell j\in G} Y^{(t)}_{h\ell j}}{\sum_{h\ell j\in G} n_{h\ell j}}
$$

for $t=1...T$, which are then used to obtain point estimates and credible intervals for cumulative incidence in Phase 1 and Phases 1 and 2 combined. The incidence proportion in Phase 2 is estimated by computing these two cumulative incidence estimates for each $t$, then taking the difference.

### 2.4 Estimating infection fatality rates outside of long-term care homes

The infection fatality rate (IFR) is a measure of the deadliness of a disease. It is defined as


$$
\text{IFR} = \frac{\text{Number of deaths from disease}}{\text{Number of infected individuals}}.
$$

The methods described in Sections 2.2 and 2.3 provide estimates of the denominator with associated uncertainty, but we still need to estimate the number of deaths in the numerator. The number of COVID-19 related deaths in Canada are publicly available, but include long-term care (LTC) residents. Our target of inference is the IFR for the “community-dwelling” Canadian population and does not apply to people living in LTC homes. The spread of COVID-19 is substantially different in LTC homes than in the general population and residents of LTC homes are particularly vulnerable to severe illness and death from infection; see Danis *and others* (2020). Indeed nearly 80$\%$ of the reported deaths from COVID-19 prior to September 2020 in Canada were in LTC homes (Samir *and others*, 2022). Modeling the spread and mortality of COVID-19 within LTC homes will require unique approaches and should be considered in a separate analysis; see the recommendations of Pillemer *and others* (2020). The Ab-C study excludes residents of LTC and thus we need to exclude this population from our numerator as well. To do this, we will extend our post-stratified mixture models to estimate the deaths outside of long-term care homes, using publicly available COVID-19 deaths data and long-term care deaths data described in Section 1.1.

In the rest of this section, we describe the extended mixture model and algorithm used to estimate IFR in this article. We start by displaying the full model with a description of each component. We then provide a directed acyclic graph (DAG) that displays the relationship between all quantities in the model. We then provide a full factorization of the posterior distribution and explain how our algorithm approximates this posterior.

#### 2.4.1 The complete model.

The full model is shown in Equations 2.3a–2.3h, followed by a description of each component. Equations 2.3a–2.3c represent the mixture model and post-stratification described previously, and will be referred to as “Module 1” of our IFR model. Equations 2.3d–2.3h represent the model extension to estimate the number of deaths outside of long-term care and will be referred to as “Module 2.” Left aligned are the model components, right aligned are the nomenclature used in the posterior factorization in Section 2.4.2.


(2.3a) \begin{align*}
\log(\boldsymbol{W}_i)|Z_i=k, x_i &\sim f_d(\boldsymbol{\mu}_k,\boldsymbol{\Sigma}_k, \nu_k) && \pi(\boldsymbol{W}|\boldsymbol{\xi},Z) \label{eq:w}\\
\end{align*}

(2.3b) \begin{align*}
\text{Prob}(Z_i=1|x_i, \beta) &= p_{h \ell j[i]}=\text{logit}^{-1}(\beta^Tx_i) && \pi(Z|\beta,x) \label{eq:Z}
\end{align*}

(2.3c) \begin{align*}
Y_{h \ell j} &\sim \text{Bin}(n_{1h\ell j}, p_{h \ell j}) && \pi(Y|\beta,x) \label{eq:Y}\\[1pt]
\end{align*}

(2.3d) \begin{align*}
D_{1\ell j} & \sim \text{Bin}(Y_{\cdot \ell j}, \eta_{\ell j}) && \pi(D|Y,\eta) \label{eq:D1}\\[1pt]
\end{align*}

(2.3e) \begin{align*}
d_{\ell j} &\sim \text{Pois}(\lambda_{1\ell j}+\lambda_{2lj}) && \pi(d|Y,\eta,\theta)\label{eq:d}\\[1pt]
\end{align*}

(2.3f) \begin{align*}
d_{2 \cdot j} &\sim \text{Pois}\left(\sum_l \lambda_{2\ell j}\right) &&\pi(d_2|\theta)\label{eq:d2}\\[1pt]
\end{align*}

(2.3g) \begin{align*}
\lambda_{1\ell j}&=Y_{\cdot \ell j} \eta_{\ell j} \label{eq:lambda1}\\[1pt]
\end{align*}

(2.3h) \begin{align*}
\lambda_{2\ell j} &= n_{2\ell j}\theta_{\ell j} \label{eq:lambda2}
\end{align*}

- Indices: $h,\ell,$ and $j$ represent education/ethnicity, age/sex, and province groups, respectively. Subscripts 1 and 2 are used to distinguish between quantities outside and within long-term care respectively.
- 2.3a: The log of the titer values, $\boldsymbol{w_i}$, of individual $i$ follow a (shifted and scaled) multivariate t-distribution, with parameters that depend on the infectious status $Z_i=k$ of that individual. $k=0$: “unvaccinated, not infected,” $k=1$: “unvaccinated, infected,” $k=2$: “vaccinated, not infected” (for Phase 2 only).
- 2.3b: an individual’s infection status, $Z_i$, depends on the infection probability corresponding to that individual’s covariate combination, $p_{h \ell j[i]}$.
- 2.3c: The number of infections in Canada with covariate combination $h\ell j$ is determined by the number of people in Canada with that covariate combination, $n_{h \ell j}$, and the probability, $p_{h\ell j}$, that a person with that covariate combination was infected.
- 2.3d: The number of deaths outside long-term care in age/sex/province group $\ell j$, $D_{1\ell j}$, depends on the number of infections in that group, $Y_{\cdot \ell j}$, and the infection fatality rate in that group, $\eta_{\ell j}$. Note that we do not attempt to estimate the deaths by education and ethnicity, which is why we sum over $h$ in $Y_{\cdot \ell j}$.
- 2.3e: The total number of COVID-related deaths in age/sex/province group $\ell j$, $d_{\ell j}$, has death rate equal to the sum of the death rates outside long-term care, $\lambda_{1\ell j}$, and the death rate inside long-term care, $\lambda_{2\ell j}$.
- 2.3f: Outside long-term care, we only know the death rates aggregated by province (the age/sex distribution is unknown). If we assume that the number of deaths outside long-term care in age/sex group $\ell$ and province $j$ follows an independent Poisson process with mean $\lambda_{2\ell j}$, then the deaths aggregated by province, $d_{2\cdot j}$, will be Poisson distributed with mean $\sum_\ell \lambda_{2\ell j}$. Note that if we knew $d_{2\ell j}$, there would be no need for Module 2.
- 2.3g: In each age/sex/province group, the mean number of deaths (death rate) outside long-term care, $\lambda_{1\ell j}$, is the product of the number of infections outside of long-term care $Y_{\ell j}$, and the infection fatality rate outside long-term care, $\eta_{\ell j}$.
- 2.3h: In each age/sex/province group, the mean number of deaths (death rate) within long-term care, $\lambda_{2\ell j}$, is the product of the number of people in Canada in long-term care $n_{2\ell j}$, and the COVID-19 death rate in long-term care, $\theta_{\ell j}$.

#### 2.4.2 Approximating the Bayesian posterior.

**Fig. 2.**
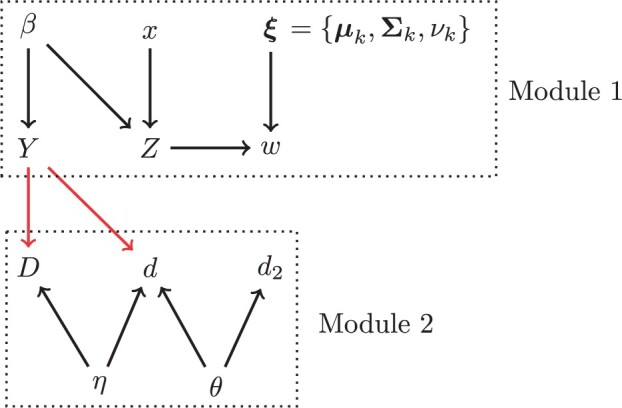
Directed acyclic graph corresponding to the model presented in equations 2.3a–2.3h, with subscripts omitted. Lower case Latin letters are known, all other terms are unknown. Module 1 is the portion of the model concerned with estimating infections. Module 2 is the portion of the model concerned with estimating deaths. The red arrows indicate a one-directional flow of information, and are the reason we are sampling from the cut distribution as opposed to the Bayesian posterior. $\beta$ is the effect of covariates, $x$, on the log(odds) of infection; $Z$ is infection status, $w$ represents titer values from the serosurvey; $\boldsymbol{\xi}$ are the parameters of the multivariate t-distributions; $Y$ is the number of infections outside of long-term care; $D$ is the number of deaths outside long-term care; $d$ is the total number of deaths by age/sex/province; $d_2$ is the number of deaths inside long-term care by province; $\eta$ is the population average probability of death given infection; $\theta$ is the COVID-19 death rate in long-term care.

Figure 2 displays the model represented in Equations 2.3a–2.3h as a DAG. Based on this DAG, the full posterior can be factored as follows:


(2.4) \begin{align*} &\pi(Y,D,\eta,\beta, \boldsymbol{\xi}, \theta, Z|x,\boldsymbol{W},d,d_2)\nonumber\\
&\propto \pi(D|Y,\eta)\pi(Y|\beta,x,d)\pi(\boldsymbol{W},d,d_2|\eta,\beta,\boldsymbol{\xi},\theta,Z,x)
\pi(\eta,\beta,\boldsymbol{\xi},\theta,Z)\nonumber\\
&=\underbrace{\pi(Y|\beta,x,d)\pi(\boldsymbol{W}|\boldsymbol{\xi},Z)\pi(Z|\beta,x)\pi(\beta)\pi(\boldsymbol{\xi})}_{\text{Module 1}}\cdot \underbrace{\pi(D|Y,\eta)\pi(d|Y,\eta,\theta)\pi(d_2|\theta)\pi(\eta)\pi(\theta)}_{\text{Module 2}}. \label{eq:full_posterior}
\end{align*}

However, sampling from this posterior poses a computational challenge, as $Y$ and $D$ are both discrete latent variables, and all three terms in $\pi(D|Y,\eta)$ are unknown. Instead, we sample from the “cut distribution” (Plummer, 2015), which is the same as Equation 2.4 but the dependence on $d$ in $\pi(Y|\beta,x,d)$ is dropped. The removal of this dependence is sometimes referred to as “cutting feedback.” Since we are not allowing our deaths data to influence our infection estimates, we are only approximating Bayesian inference when computing IFR. The cut distribution has been shown to give more sensible results than the full posterior in some scenarios where certain portions (modules) of the model are misspecified, or data quality is poor (Lunn *and others*, 2009). It is important to note that our serosurvey data are very high quality individual level data, but our deaths data are partially imputed and is from an unofficial source. The cut model allows us to base our estimates of incidence solely on the serosurvey data (and census data), while still utilizing all data sources to estimate IFR. We sample from the cut distribution using the following two step algorithm:

(1) We first sample from the joint posterior of the parameters in the first module:


\begin{align*}
\pi(Y,\beta, \boldsymbol{\xi}, Z|x,\boldsymbol{W})& \propto \pi(Y|\beta,x)\pi(\boldsymbol{W}|,\boldsymbol{\xi},Z)\pi(Z,\boldsymbol{\xi},\beta) \\
&=\pi(Y|\beta,x)\pi(\boldsymbol{W}|\boldsymbol{\xi},Z)\pi(Z|\beta,x)\pi(\beta)\pi(\boldsymbol{\xi}),
\end{align*}

which is the same as the Module 1 portion of Equation 2.4 but with the dependence of $d$ dropped in the first term. We sample from this distribution by obtaining $T$ (post burn-in) posterior samples of each parameter using $\pi(\beta, \boldsymbol{\xi}, Z|x,\boldsymbol{W}) = \pi(\boldsymbol{W}|\boldsymbol{\xi},Z)\pi(Z|\beta,x)\pi(\beta)\pi(\boldsymbol{\xi})$ as a target distribution in MCMC. We then draw a sample, $Y^{(t)}$, from $\pi(Y|\beta^{(t)},x)$ for $t=1...T$.

2) For each $t=1...T$, we use MCMC to obtain 1 post burn-in sample from the posterior of Module 2. To do this, we first obtain one post burn-in sample using $\pi(d|Y^{(t)},\eta,\theta)\pi(d_2|\theta)\pi(\eta)\pi(\theta)$ as the target in MCMC for each $t=1...T$. We then sample $D^{(t)}$ from $\pi(D|Y^{(t)},\eta^{(t)})$ for $t=1...T$.

We used this algorithm for both Phase 1 and Phase 2 data, obtaining $T$ samples of $(Y_{\cdot \ell j}, D_{1\ell j})$ from $\pi_\text{cut} (Y,D)$. We then estimate IFR by computing samples from $\pi_\text{cut}(\text{IFR}_G)$ for any subgroup of Canadians $G$ outside of long-term care:


(2.5) \begin{equation*}
\label{eq:IFR}
\text{IFR}^{(t)}_{G} = \frac{\sum_{\ell j \in G}D^{(t)}_{1 \ell j}}{\sum_{\ell j \in G}Y^{(t)}_{\cdot \ell j}}
\end{equation*}

for each $t=1...T$. We can then compute point estimates with uncertainty for all of Canada, and any age/sex/province combination that we so please. We compute the $\text{IFR}_{G}$ for various age/sex/province combinations using univariate and bivariate models to estimate the denominators for the Phase 1 data, and the multivariate model for Phase 1 and 2 combined. We do not attempt to estimate IFR by education/ethnicity, so we sum over $h$ in $Y_{\cdot \ell j}$.

Since individuals who were likely to be positive in Phase 1 were also likely to be positive in Phase 2, estimating incidence and deaths just based on Phase 2 data will also include people who were likely infected in Phase 1. In order to estimate the new infections and deaths (and as a result, IFR) in just Phase 2, we found posterior samples of $Y$ from the multivariate model and subtracted the posterior samples from the bivariate model to get the denominator. The same was done for the deaths $D$ for each posterior sample, allowing us to calculate IFR for any subgroup we desire.

### 2.5 Priors

In all three mixture models, a weakly informative prior of $N(0,1)$ was used for each $\beta$. This will stabilize estimates in groups with a small amount of data, and have little effect on those that have a lot of data. A weakly informative penalized complexity prior was put on the degrees of freedom in all three models (see Appendix A). In the multivariate cases, informative priors were used to overcome well-known computational challenges of fitting Bayesian mixture models as noted in the Stan documentation (Betancourt, 2017). We describe our informative priors and their justifications in detail in Appendix D.1. In the reproducible example that we provide in the Supplementary material available at *Biostatistics* online, we show that our results are not too sensitive to “mis-specified” informative priors on the mixture components. We also note that it is primarily the estimation of $\beta$’s that influence the results of this article. A weakly informative prior was used on $\Sigma$ as recommended by Section 1.13 of the Stan User’s Guide (Stan Development Team, 2021). A complete list of priors for all models is presented in Appendix D.

### 2.6 Inference

Each model was run using No-U-Turn sampling, a form of Hamiltonian Monte Carlo that is readily available in the Stan software (Carpenter *and others*, 2017,^2021^). Four chains with 1000 iterations, with the first half being warmup, were used for each model component. Traceplots were used to visually assess convergence of Markov chains, alongside values of $\text{Rhat}<1.01$ confirming an appropriate amount of mixing (Vehtari *and others*, 2021). Point estimates are taken to be the 50th percentile of the (approximate) posterior distributions, and credible intervals (CrI’s) are computed using the 2.5th and 97.5th quantiles.

## 3. Results

### 3.1 Univariate model—Phase 1

**Fig. 5.**
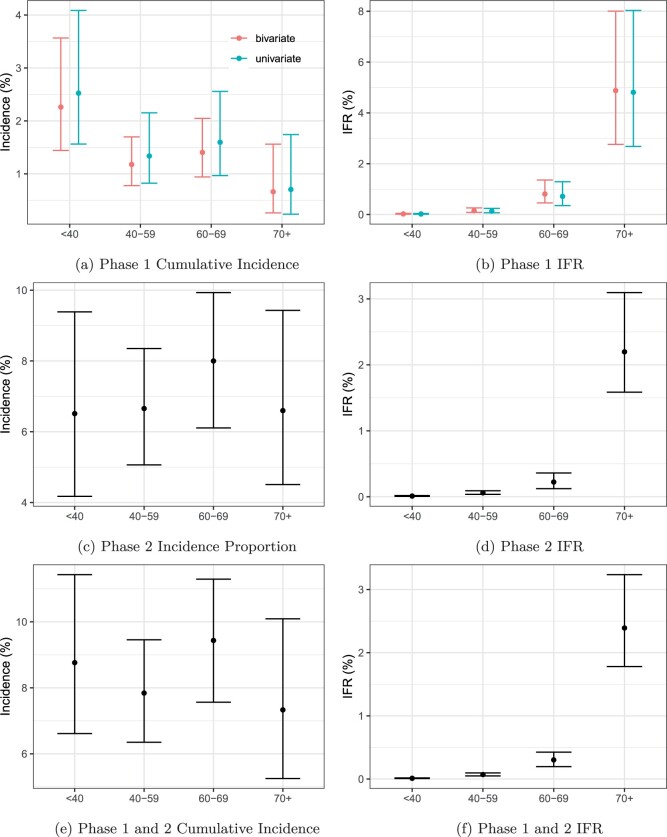
Incidence/IFR by age (years) for each time period. Posterior medians are used as point estimates, and the 2.5th and 97.5th posterior quantiles define the error bars.

Estimated cumulative incidence and IFR by age group is presented in Figure 5. Using the univariate model, the overall estimated cumulative incidence in Phase 1 (February–Sept 2020) is 1.79$\%$ (95$\%$ CrI: 1.21–2.66), which is similar to the estimate presented in Tang *and others* (2022) of 1.9$\%$ (95$\%$ CI: 0.7–4.7). Using this model for the denominators in the IFR calculation leads to an estimated infection fatality rate of 0.35$\%$ (95$\%$ CrI: 0.24–0.52) for all Canadians outside of long-term care homes. This is, again, consistent with the estimates presented in Tang *and others* (2022) of 0.373 (95$\%$ CI: 0.153–1.024).

When we look at the age distribution of cumulative incidence, we see a general downward trend with increasing age, with estimates for the age group 70+ being the smallest at 0.71$\%$ (95$\%$ CrI: 0.24–1.74). However, the credible intervals all overlap which suggests that incidence is similar between age groups. We see an upward trend in IFR with increasing age, with non-overlapping credible intervals. This is to be expected, as COVID-19 is now known to be much deadlier in older populations (Williamson *and others*, 2020).

A plot of the two univariate t-distributions is shown in Figure 1. Notice that the density plot for the positive group has mass to the left of the cutoff used by Tang *and others* (2022), and the negative group has mass to the right of the cutoff. Large values of titers ($>$2) will show high probability of SARS-CoV-2 incidence from our model, but this is not true for titer values around $0.5$. If these values had been discretized using a fixed cutoff, participants with very large titer values would be indistinguishable from those with values of $\approx 0.5$, thus would have the same probability of being false positives. Although this univariate case works well to demonstrate our method, we will use the results from the bivariate model when computing estimates for Phase 1.

### 3.2 Bivariate model—Phase 1

Figure 5 presents estimated cumulative incidence and infection fatality rates for the bivariate model in Phase 1 using both SmT1 titers. The overall cumulative incidence for Canada was 1.60$\%$ (95$\%$ CrI: 1.15–2.23). This point estimate is somewhat consistent (slightly lower) with the univariate results, with a smaller credible interval. This is reassuring, since our uncertainty should decrease as more data is used in the model. Our Phase 1 estimates are comparable with the estimate for seroprevalence in Canada from O’Driscoll *and others* (2021) of 1.4$\%$ (CI: 1.16–1.68, as of September 1st 2020). The estimated overall infection fatality rates for residents outside of long-term care homes was 0.39$\%$ (95$\%$ CrI: 0.27–0.56), which is also consistent with our univariate results. We will use the bivariate results for Phase 1 going forward.

When broken down by age, we see very similar trends in both cumulative incidence and IFR as with the univariate model. We also see slightly reduced uncertainty in all age groups, which is to be expected since we are adding more information (an extra titer value) into the model. The decrease in uncertainty is small, suggesting that the additional assay did not provide much additional information when predicting infection. We can investigate which titer value had more influence on the probability of infection by computing


$$
\text{Prob}(Z_i=1|\boldsymbol{w}_i)=\frac{\text{Prob}(\boldsymbol{w}_i|Z_i=1) \text{Prob}(Z_i=1)}{\text{Prob}(\boldsymbol{w}_i)}.
$$

That is, we compute the probability of infection given the titer values, which are easily computed based on results from (2.2).

**Fig. 3.**
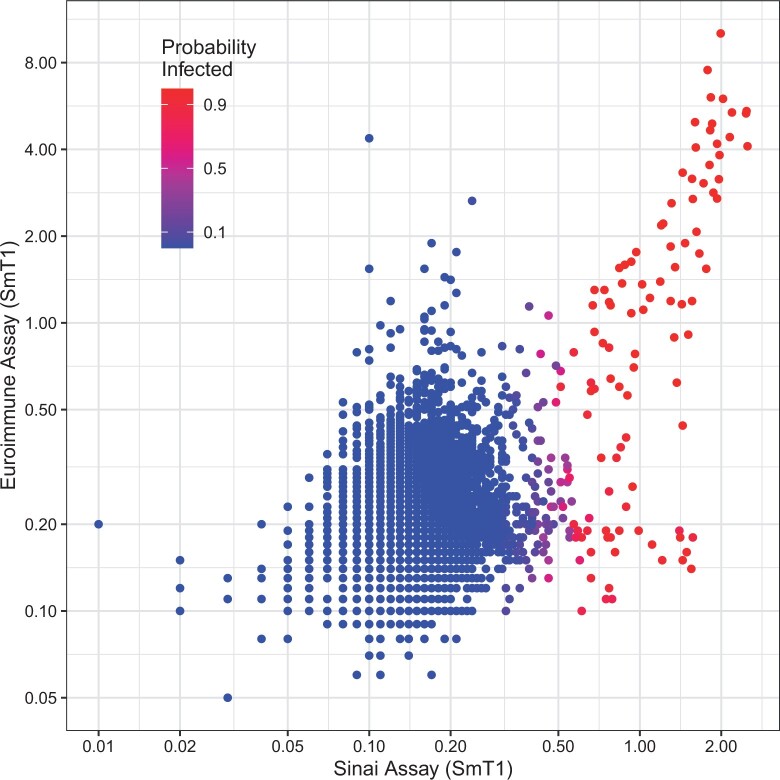
Probability of infection given each individual’s titer values using the bivariate mixture of t-distributions in Phase 1. Each dot represents a participant in the Ab-C study. On the x-axis is the titer value that was used in the univariate model. On the y-axis is an second SmT1 protein assay. A red dot indicates that this model predicts a high probability of infection, with blue being a low probability of infection, and purple being indeterminate.

Figure 3 shows the probability of infection given each individual’s titer values using the Bivariate mixture of t-distributions. Our model seems to “trust” the Sinai titer value more, given that it predicts a high probability when the Sinai value is high, even if the Euroimmune titer value is low. Our model seems to be indeterminate around the cutoff (Sinai titer value $\approx 0.5$) that was chosen by Tang *and others* (2022), which implies some agreement between the two methods.

### 3.3 Trivariate model—Phase 2

Estimates of cumulative incidences and infection fatality rates in Phase 2 are presented in Figure 5(c) and (d). Using a trivariate mixture of t-distributions with three latent groups and post-stratification, the estimated incidence proportion in Phase 2 was 6.81$\%$ (95$\%$ CrI: 5.35–8.42). This is obviously much higher than our estimates in Phase 1, which is to be expected. The estimated infection fatality rate in Phase 2 was 0.31$\%$ (95$\%$ CrI 0.25–0.39), which is slightly lower than Phase 1. This is comparable, but slightly lower than other estimates for Canadian IFR ($\sim$ 0.65$\%$ from O’Driscoll *and others* (2021)), which is unsurprising since our study excluded those in nursing homes.

The incidence proportion in Phase 2 was comparable across age groups, with the IFR again trending upwards with age. In Phase 2, see that each age category had a lower IFR than Phase 1. Our estimates of IFR by age were highly comparable to international estimates (see Table S3 of O’Driscoll *and others* (2021)).

**Fig. 4.**
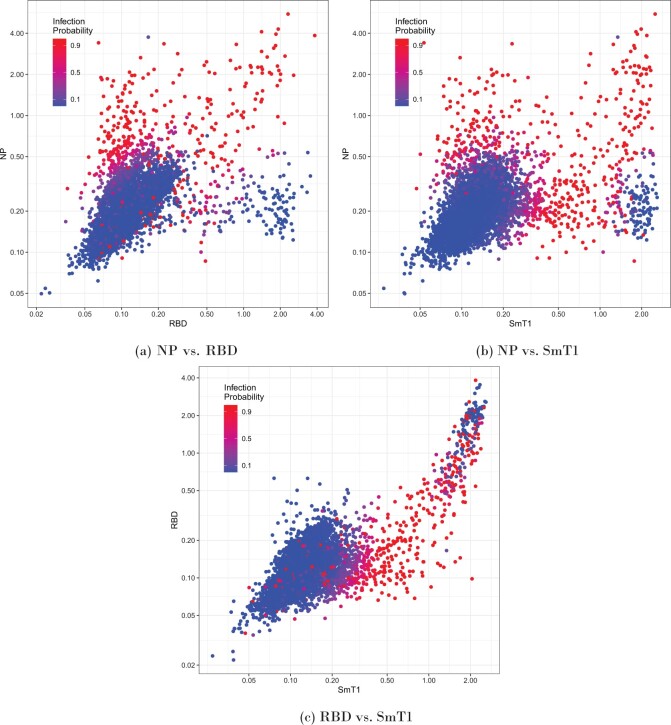
Probability of infection given each individual’s titer values using the trivariate mixture of t-distributions in Phase 2. A red dot indicates that this model predicts a high probability of infection, with blue being a low probability of infection, and purple being indeterminate. In theory, participants who have never been infected or vaccinated should have low values for all three titers. Vaccinated, but never infected individuals should have high SmT1 and RBD, but low NP, and infected individuals have high values for all three.

The cumulative incidence and IFR’s for Phase 1 and Phase 2 combined are shown in Figures 5(e) and (f). The cumulative incidence estimate is 8.41$\%$ (95$\%$ CrI: 7.04–9.92), with an IFR of approximately 0.31$\%$ (95$\%$ CrI: 0.27–0.37). The patterns in incidence and IFR by age are highly similar to those in Phase 2 alone. The probabilities of infection given the titer values of each participant are shown in Figure 4. Since our outcome is three-dimensional, three separate plots are required. Blue dots in the bottom right corner of Figures 4(a) and (b), and the top right corner of Figure 4(c), identify participants that are likely showing immunity due to being vaccinated, as vaccinated individuals should be low on NP and high on the other two. We see that our model tends to “trust” the NP and SmT1 titers more when predicting infection. People who are high on NP or SmT1 tend to have higher probabilities, while people with only high RBD values tend to have a low probability of infection.

### 3.4 Cumulative incidence and IFR by province

One advantage to the methods presented in this article, is that once we have posterior samples for infections and deaths outside of long-term care, we can break the results down by any covariate combination that we so please. Figure B2 shows the cumulative incidence and infection fatality rates by province in both phases. In Phase 1, Ontario had the highest point estimate for cumulative incidence, and Quebec had the highest IFR. Our estimated IFR in Ontario was 0.27$\%$ (95$\%$ CrI: 0.19–0.41) in Phase 1, which is much lower than the estimate given by Public Health Ontario at the time (2.8$\%$ as of May 17, 2020 (Public Health Ontario, 2020)). Although these numbers are not directly comparable, as our estimates do not include people in nursing homes, this likely doesn’t account for all of the disparity. Public Health Ontario’s number was estimated based on IFR numbers obtained using individual-level data from China (Verity *and others*, 2020) and was adjusted to match the age distribution of Ontario. We therefore remain somewhat skeptical of the numbers presented in Public Health Ontario (2020). When comparing our overall estimate to the estimate in Verity *and others* (2020) (0.657$\%$, CI 0.389–1.33), our number is much more comparable.

In Phase 1, Quebec had a very high reported number of deaths, which was not proportional to the number of long-term-care home deaths, resulting in a high IFR. In Phase 2 Quebec’s incidence went up substantially, while the IFR dropped significantly. In Phase 2, the credible intervals for both cumulative incidence and IFR overlap between provinces.

**Fig. B1.**
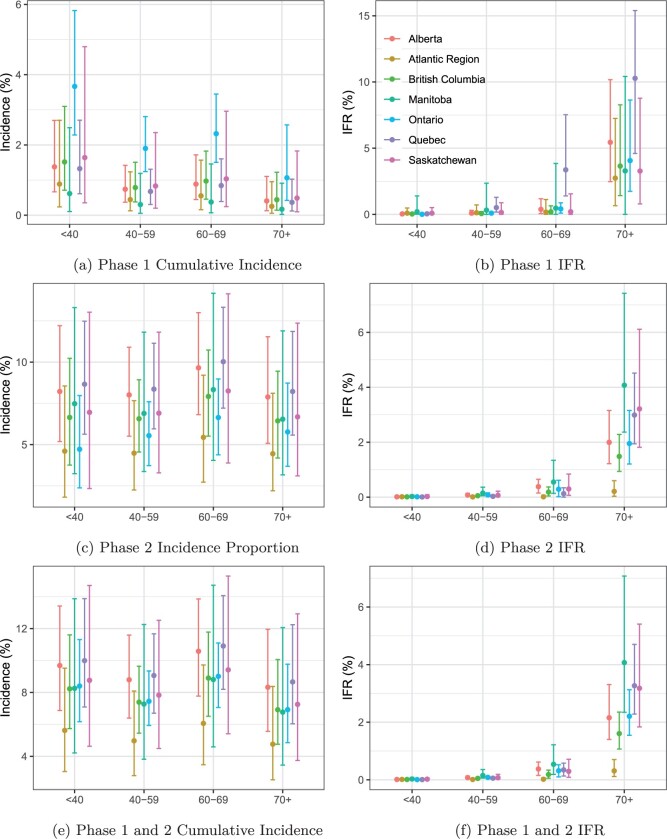
Incidence/IFR by age (years) in each province. Posterior medians are used as point estimates, and the 2.5th and 97.5th posterior quantiles define the error bars.

**Fig. B2.**
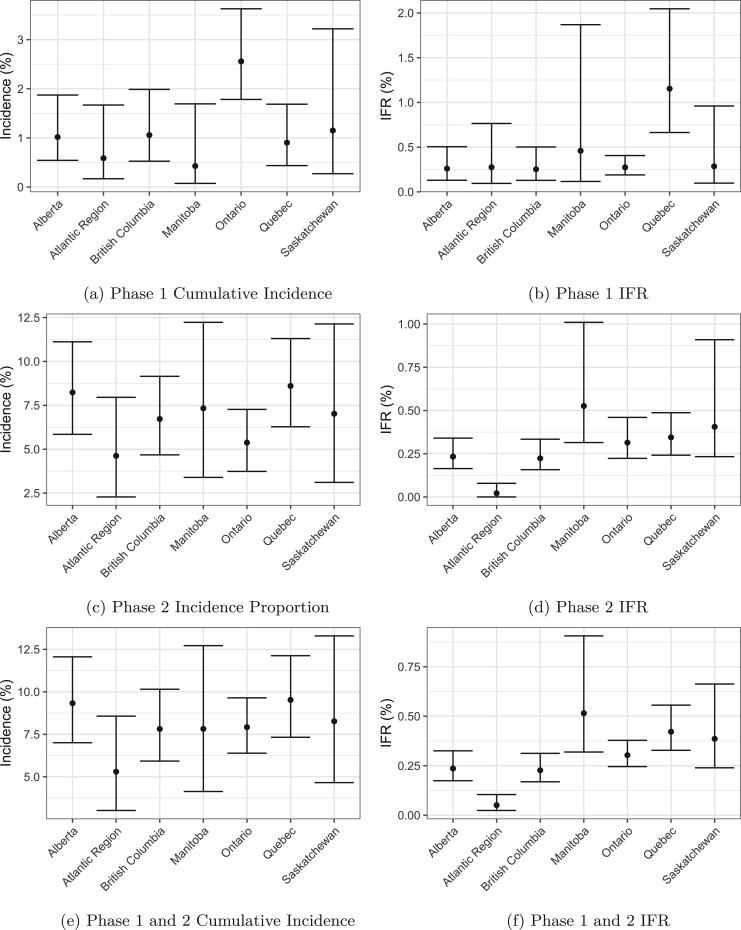
Incidence/IFR by province. Posterior medians are used as point estimates, and the 2.5th and 97.5th posterior quantiles define the error bars.

Estimates by age group in each province are shown in Figure B1. In all provinces, incidence in Phase 1 was highest in 18- to 39-year-old, and lowest in 70+ year old. With the exception of Alberta, this pattern did not hold in Phase 2, as incidence seems to be less predictable as a function of age. In each province and phase, IFR reliably trends upwards with age.

Estimates of incidence by ethnicity in each province are shown in Appendix C. In both phases, the white and indigenous groups have comparable incidences in each province. The “not white or indigenous” group (NWoI) has relatively high incidence in Ontario and British Columbia in both phases, and low incidence in the Atlantic region and Saskatchewan in Phase 2. Note that estimates of IFR are not reported by ethnicity, as we do not have (even aggregate) COVID-19 deaths data by ethnicity.

## 4. Discussion

In this article, we developed an approximate Bayesian approach to estimate cumulative incidence and IFR using a multivariate mixture of t-distributions. We used data from the Ab-C serosurvey to estimate the probability that individuals were infected with COVID-19 based on their titer values and covariate combinations, and used post-stratification to generalize our results to the Canadian population that resides outside of long-term care. Our Phase 1 cumulative incidence estimates were slightly lower than previous estimates based on fixed cutoffs. Our Phase 2 estimate was higher than the one in the literature. Furthermore, our method accounts for uncertainty in both the number of infections and the number of deaths, and is essentially a cut model where we do not allow the deaths data to affect the estimation of the number of infections.

Estimates of incidence by age do not show any noteworthy patterns other than a slight upward trend in Phase 1. In both Phase 1 and Phase 2, IFR increased with age. Furthermore, IFR was higher in Phase 2 than Phase 1 in each age group, although the overall IFR was the same.

The main strength of our approach is that it uses the exact titer values as outcomes in our model, as opposed to a discretized version which discards information. Furthermore, we can leverage multiple titer values in a multivariate model to improve estimated probabilities of infection, while being able to differentiate between previously infected and vaccinated individuals. An additional strength of our study is that error is correctly accounted for in both the calculation of the number of infections and deaths outside long-term care, and consequently, IFR. We have not considered under-reporting of COVID-19 deaths, and we acknowledge this could be a potential issue. One way to accommodate this would be to make an assumption that a known proportion of COVID-19 deaths go unreported and include draws of unreported deaths in each posterior sample of the IFR. In the absence of information of what this proportion should be, we have treated the reported death counts as correct with the caveat that the estimated IFRs only refer to deaths directly attributed to COVID-19.

A methodological limitation of this study is that we are assuming that both the infected and uninfected groups follow a multivariate t-distribution. This may not be the most appropriate distribution for these data, and perhaps a distribution that allows for skewness may be more appropriate. Although our model makes no direct assumption about sensitivity and specificity, these two quantities are directly related to the length of the tails of the t-distributions for any given cutoff. However, the parameters of the multivariate t-distribution are estimated from the data, so our method is analogous to a non-discretized version of the methodology presented in Gelman and Carpenter (2020), where sensitivity and specificity are parameters to be estimated in the model.

**Fig. G1.**
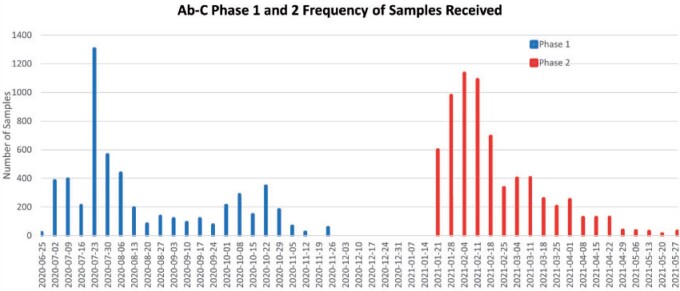
Distribution of dates of samples received for Phase 1 and Phase 2.

A second limitation is that some responses to the survey happened before the end of the survey, such that they could have returned a “negative” dry blood spot sample and subsequently gotten infected. This would lead to slightly underestimating incidence (overestimating IFR). On the other hand, there is a time lag between infection and death, so if we counted infections up until the end of September 2020, then those infected people could experience death several weeks later and not be recorded. However, given that the vast majority of participants returned their blood samples study more than two weeks prior to each Phase’s end date (see Figure G1), we figured that accounting for this time lag was not necessary.

A third limitation of our methodology is that we were unable to incorporate information regarding Phase 1 infection probabilities (from SmT1 protein) into our Phase 2 estimates of incidence. Although Phase 1 and Phase 2 SmT1 protein titer values are not directly comparable (due to the assays being calibrated slightly differently), we recognize that there is some potential to treat the SmT1 titer longitudinally from Phase 1 to Phase 2. However, we figured that this would require a drastic reworking of our current model and inference framework, and thus we deemed it out of the scope of this article. The potential consequence of this is a slight underestimate of cumulative incidence at the end of Phase 2, as some “infected” individuals in Phase 1 may be overlooked by solely looking at Phase 2 titer values (see Appendix E for a sensitivity analysis and discussion), with waning being one potential cause. However, Tang *and others* (2022) show that roughly 80$\%$ of people retain their “seropositivity” status from Phase 1 to Phase 2. The exploratory analysis presented in Appendix F suggests that waning may not be a large issue. It is also possible that people who were infected in Phase 1 were reinfected in Phase 2. Reinfected individuals will likely have titer values that are exceptionally high, which would affect our estimates of the parameters for the mixture distributions. This also would make the interpretation of incidence murky, as reinfected people only count as one infection. We suspect this to be more of an issue when estimating incidence/IFR at later dates, as the number of reinfected individuals in our study is expected to be very small.

A direction for future work will be to apply these methods to upcoming Phase 3 and Phase 4 data that includes a much larger vaccinated population, as well as breakthrough infections in people who have been vaccinated. Furthermore, we will have to account for reinfections as the populations’ immunity wanes and new variants emerge. This could involve a longitudinal mixture model or Hidden Markov Model. Furthermore, an improved serosurvey design and associated statistical methodology that allowed for estimation of incidence (and consequently, IFR) in real-time would be an ambitious and highly interesting area of future research.

This study only looks at humoral immune response, but cellular immunity also plays an important role in the immune response to SARS-CoV-2. Other studies have evaluated the effects of T-cell response in infected people (Guo *and others*, 2022,^2022^). An interesting line of future work would be to develop similar methods to incorporate T-cell response data into estimates of incidence and IFR.

Although we focused on SARS-CoV-2 infections and deaths in this paper, the methods presented can be applied to a variety of outcomes for any infectious disease of interest in which serosurvey data are available. There are plenty of potential extensions to this model that can be implemented to suit a variety of problems in epidemiology and biostatistics.

## Supplementary Material

kxad003_Supplementary_Data

## Figures and Tables

**Fig. B3.**
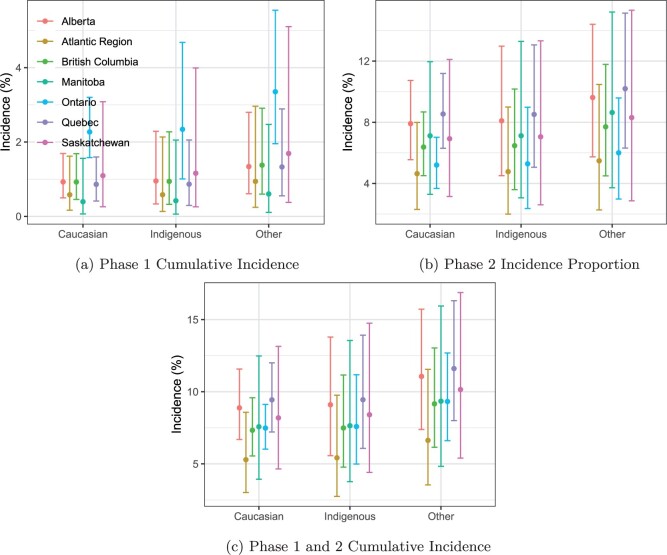
Incidence by ethnicity in each province. Posterior medians are used as point estimates, and the 2.5th and 97.5th posterior quantiles define the error bars.

**Table D1.** *Priors used in Phase 1 univariate model*

Parameter	Prior
$\mu_0, \mu_1$	$N(0,10)$
$\sigma_0,\sigma_1$	$N_+(0,10)$
$\beta$	$N(0,1)$
$\nu_k$	$\text{Prob}(\nu > 10) = 0.5$

**Table D2.** *Priors used in Phase 1 bivariate model*

Parameter	Prior
$\mu_0$	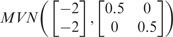
$\mu_1$	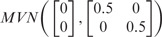
$\beta$	$N(0,1)$
$\nu_k$	$\text{Prob}(\nu > 10) = 0.5$
$\boldsymbol{\Sigma}_k = \text{diag}(\tau) \times \Omega \times \text{diag}(\tau)$	
$\tau$	$\text{Cauchy}_+(0,1)$
$\Omega$	$\text{LKJCorr}(2)$

**Table D3.** *Priors used in Phase 2 mixture model*

Parameter	Prior
$\mu_0$	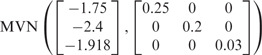
$\mu_1$	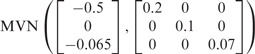
$\mu_2$	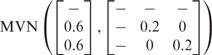
$\beta$	$N(0,1)$
$\nu_k$	$\text{Prob}(\nu_k > 30) = 0.5$
$\rho$	$N_+(0.015,0.0025)$
$\boldsymbol{\Sigma}_k = \text{diag}(\tau) \times \Omega_k \times \text{diag}(\tau)$	
$\tau$	$\text{Cauchy}_+(0,1)$
$\Omega_k$	$\text{LKJCorr}(0.5) \prod_c N(c|m_c, s_c)$

**Table D4.** *Priors used in deaths module (Section 2.4.2)*

Parameter	Prior
$\eta$	$N_+(0.004,0.05)$
$\theta$	$N_+(0.01,0.1)$

**Fig. E1.**
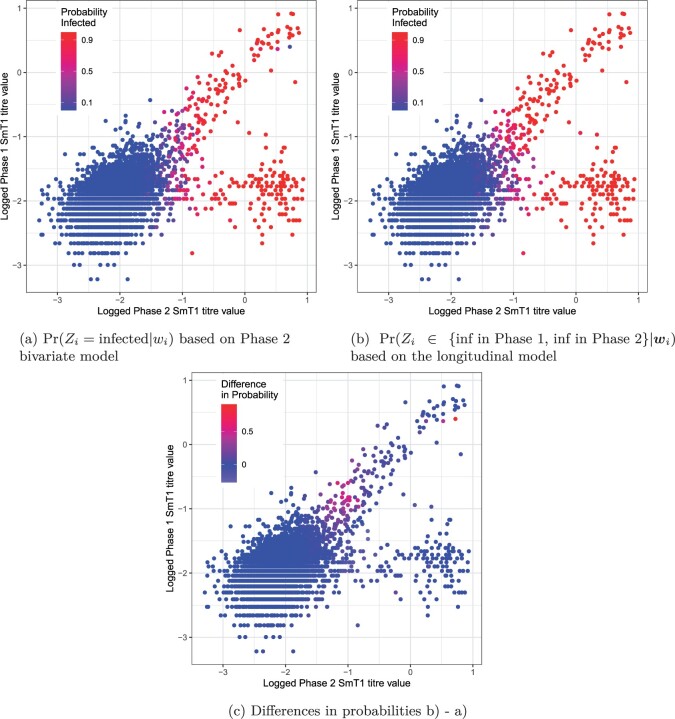
Comparing infection probabilities between the bivariate (Phase 2) and longitudinal (Phases 1 and 2) models. In (a) and (b), blue points indicate low probability of infection, while red indicates a high probability of infection. In (c), blue indicates agreement between the two models, while a more red color indicates a high estimated infection probability from the longitudinal model.

**Fig. F1.**
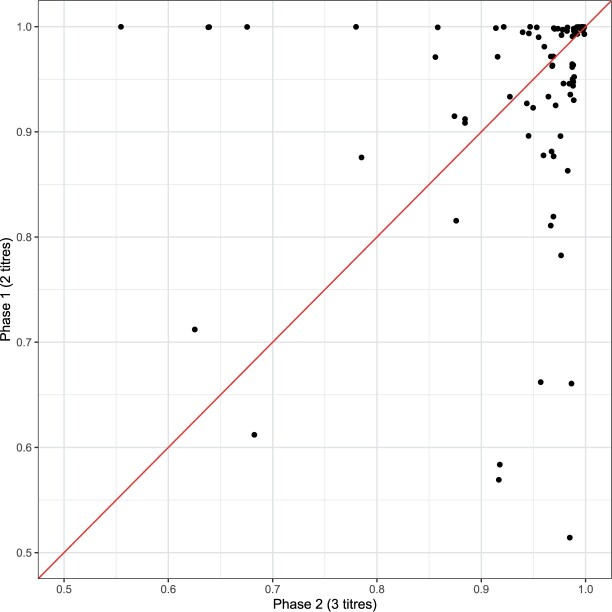
Phase 1 versus Phase 2 predicted probabilities for participants who had large predicted probabilities in Phase 1. Points above the red line indicate that Phase 1 predicted probability was higher.

## Software

The serosurvey data used in this paper is highly confidential and cannot be shared publicly. We have supplied a reproducible example using simulated serosurvey data to demonstrate how our method is implemented: https://github.com/JustinJSlater/AbC-Bayesian-Mixture–Reproducible-Example

## A. Penalized complexity prior on degrees of freedom ν

As mentioned in Section 2.2.1, we noticed that a Normal distribution is likely not heavy-tailed enough to accurately model the log(titer) of the non-infected group. The t-distribution adds a degrees of freedom parameter, $\nu$, which controls how heavy-tailed the t-distribution is relative to the Normal distribution. The t-distribution reduces to a Normal distribution as $\nu \to \infty$. Therefore we can view $\nu$ in this case as a parameter that adds complexity to a base model, the Normal model. The closer $\nu$ is to 1, the more “complex” the model is. Simpson *and others* (2017) outlines a framework for penalizing model component complexity as a function of the distance to a base model. We used a penalized complexity (PC) prior on $\nu$ that will encourage $\nu$ to be large (closer to the Normal model) unless there is appropriate evidence in the data.

Rather than putting a prior on $\nu$ itself, Simpson *and others* suggest putting a prior on the root Kullback–Leibler (KL) distance:

(A.1)
\begin{equation*}\label{eq:PC}
\delta(\nu) = \sqrt{2\cdot D_\text{KL}[t_\nu(\boldsymbol{\mu}, \boldsymbol{\Sigma}) ||\mathcal{N}(\boldsymbol{\mu}, \boldsymbol{\Sigma})]},
\end{equation*}

where $t_\nu$ and $\mathcal{N}$ denote the multivariate t and normal densities respectively, and $D_\text{KL}$ is the KL divergence. Note that the shifting ($\boldsymbol{\mu}$) and scaling ($\boldsymbol{\Sigma}$) parameters cancel out, and hence $D_\text{KL}$ is only a function of $\nu$ (Villa and Rubio, 2018). Unfortunately, $D_\text{KL}$ in Equation (A.1) has no closed form that the authors are aware of, so we computed it numerically as described in Appendix A.

Villa and Rubio (2018) showed that the KL divergence between two $d$-dimensional multivariate-t distributions, $f(\boldsymbol{x}|\boldsymbol{\mu},\boldsymbol{\Sigma}, \nu)$, and $f(\boldsymbol{x}|\boldsymbol{\mu},\boldsymbol{\Sigma}, \nu'),$ is

$$
\log \frac{K(d,\nu)}{K(d,\nu ')} - \frac{\nu + d}{2} \text{E}_{f} \Big[\log \Big(1+ \frac{\boldsymbol{x}^T\boldsymbol{x}} {\nu}\Big) \Big ] + \frac{\nu'+d}{2} \text{E}_{f} \log \Big (1+\frac{\boldsymbol{x}^T\boldsymbol{x}}{\nu'}\Big)
$$

where

$$
K(d,\nu) = \frac{\Gamma(\frac{\nu + d}{2})}{\Gamma (\frac{\nu}{2})\sqrt{(\pi \nu)^d}}
$$

The two expectations are shown to be

\begin{align*}
\text{E}_{f} \Big[\log \Big(1+ \frac{\boldsymbol{x}^T\boldsymbol{x}} {\nu}\Big) \Big ] &= \Psi \Big ( \frac{\nu + d}{2}\Big) - \Psi\Big(\frac{\nu}{2}\Big)\\
\text{E}_{f} \Big[\log \Big(1+ \frac{\boldsymbol{x}^T\boldsymbol{x}} {\nu'}\Big) \Big ] &= K(d,\nu) \frac{\pi^{\frac{d}{2}}}{\Gamma (\frac{d}{2})} \int^\infty_0 \Big (1+ \frac{t}{\nu}\Big)^{-\frac{\nu + d}{2}}t^{\frac{d}{2}-1} \log \Big ( 1+\frac{t}{\nu'}\Big ) dt
\end{align*}

Meaning that the $d$-dimensional integral can be reduced to one-dimensional integral. Since we are interested in the KLD between a multivariate T and a multivariate normal, we substitute $\nu'=200$, and compute this integral numerically as a function of $\nu$. We then approximate the distance, $\delta (\nu) = \sqrt{2 \cdot \text{D}_\text{KL}}$ with a polynomial. For example, $\delta (\nu)$ for the bivariate model was $\delta (\nu) \propto \nu^{-1.3}$. We then say that

$$
\pi(\delta (\nu)) \sim \text{exp}(\lambda)
$$

with $\lambda = -\log(\alpha)/\delta (U),$ where $\alpha$ and $U$ are chosen such that our prior belief is that there is a 50$\%$ chance that $\nu$ is greater than 30.

## D. Prior distributions

### D.1 Phase 2 model prior justification

As mentioned in the main text, we require informative priors for computational reasons. In this Section, we justify our choices of informative priors for the Phase 2 trivariate model. We note that these priors are not very sensitive to

- $\mu_0$ corresponds to the means of the “not infected” group. The first element of $\mu_0$ corresponds to the mean NP titer values in “not infected individuals”. Alongside the NP titer values collected from the survey, the lab also provided us with “control” samples of known negatives. We found that the vast majority of the control samples fell between $-$2.5 and $-$1 on the log scale. Therefore we are very confident that the *mean* of NP titer values from “not infected” people should be in this range. Therefore, we applied the conservative but informative prior $N(-1.75, 0.25)$. Similar reasoning was used for the prior on the second element of $\mu_0$, corresponding to the mean of RBD titer values in “not infected” people.
- When setting priors for the “not vaccinated, not infected” and “infected” groups based on Smt1 titer values, we used the corresponding posterior distributions from Phase 1. Although the tests are calibrated slightly differently, and there will be a small amount of waning between phases, we do expect these values to be somewhat similar.
- To determine the posterior of the mean of the infected group for NP titer values (first element of $\mu_1$), we consider the fact that any titer value above mean+3SDs is likely a previous infection (this is how the cutoff was chosen in Tang et al.). We then ensure that the bulk of the prior distribution for the positive N group was above this value, with some overlap. We used similar reasoning for the RBD positive group.
- To determine the prior for the mean RBD/SmT1 titer values in the vaccinated groups, we used similar reasoning as above, trying to ensure that the prior has most of it’s mass above that of the infected group’s with some overlap.
- We used a weakly informative prior for $\Omega_k$ using the LKJ distribution with shape = 0.5. This provides a roughly uniform distribution across positive-semidefinite $3\times3$ matrices. We then add additional information for each off-diagonal by multiplying by normal densities. For instance, if we suspect that the correlation between two parameters should be positive (i.e., off-diagonal element $c$ of $\Omega_k$ is positive), we multiply the prior for $c$ by $N(c|0.5,0.2)$ which gently encourages the correlation to be positive but still has mass below 0.

## E. Longitudinal sensitivity analysis

As mentioned in Section 4, there is potential for these data to be used in a longitudinal way, as roughly 6300 survey participants had titer values in both Phase 1 and Phase 2. SmT1 titer values are measured in both phases, while RBD and NP are only available in Phase 2. Thus in this section, we wanted to examine the potential effects of ignoring the longitudinal nature of these data. In the analysis of the main paper, we use only titers from Phase 2 to determine cumulative incidence in Phase 2. Can we better estimate cumulative incidence in Phase 2 by incorporating Phase 1 measurements into the model? Due to computational/methodological reasons, we can not answer this question perfectly. However, we fit models that can potentially provide insight into the effect of not looking at the data longitudinally.

For this analysis, we made the following simplifications/assumptions:

- Excluded anyone who claimed to have been vaccinated. The addition of a vaccinated group causes computational challenges when fitting the longitudinal mixture model, due to the additional group.
- We do not consider covariates (age, sex, etc.), as these are mainly used for post-stratification.
- We only consider one titer in Phase 1 (SmT1 Sinai assay) and two titers in Phase 2 (SmT1 and RBD). Note that the exclusion of the NP titer value will cause underestimation of seroprevalence by $\approx 2\%$. This simplification is necessary as these mixture models become hard to fit with more than three titers without unjustifiably strong prior information.

In this analysis, we fit two models. The first is a bivariate mixture model using only the titers available from Phase 2 (SmT1 and RBD). This is analogous to what we did in our paper (minus the third titer value, NP). We then compare this to a “longitudinal” model, which a trivariate mixture model using all three titers (one from Phase 1, two from Phase 2), and three mutually exclusive infection groups: “not infected,” “infected in Phase 1,” and “infected in Phase 2.”

We compared the predicted probabilities of infection for each survey participant based on the bivariate model and the longitudinal model. Figure E1a displays $\text{Pr}(Z_i = ``\text{previously infected}''|\boldsymbol{w_i})$ for the model fit using solely Phase 2 SmT1 and RBD titer values. Figure E1b shows probabilities of previous infection, given all three titer values, $\text{Pr}(Z_i \in \{ ``\text{infected in Phase 1, infected in Phase 2}''\} | \boldsymbol{w}_i)$, for the longitudinal model. To reduce the total number of plots, we display these probabilities on plots without an RBD axis. We can see that the longitudinal model predicts higher infection probabilities for a small subset of individuals who were marginally high on SmT1 in both phases. These individuals are highlighted in Figure E1c, where we plot the difference in predicted probabilities between models for each individual. This plot indicates that the non-longitudinal model may be missing some infected individuals when we do not consider the Phase 1 titer. The estimated prevalence in the cohort is 4.5$\%$(95$\%$ CrI: 3.9–5.3) based on the bivariate model, and 4.9$\%$ (95$\%$ CrI: 4.1–6.4) based on the longitudinal model. However, of the 8 people who had very substantial differences in their predicted probabilities ($>$50$\%$), 6 of them had high NP titer values in Phase 2, thus the third titer that we excluded would potentially make up for part of the disparity.

In summary, when estimating infection probabilities in Phase 2 based solely on Phase 2 titers, we tend to slightly underestimate average infection probabilities when compared to the longitudinal model: 4.5$\%$(95$\%$ CrI: 3.9–5.3) versus 4.9$\%$ (95$\%$ CrI: 4.1–6.4). However, it appears that some people “missed” by the nonlongitudinal model would have been captured by the NP titer value in Phase 2. Hence, the overall effect of not considering Phase 1 titer values when estimating cumulative incidence in Phase 2 is likely small, but measureable.

A longitudinal model may help improve incidence estimation prospectively. However, adding additional titer values from either phase would also likely improve incidence estimation. Without using all titer values from both phases, it is hard to determine the effects of ignoring the longitudinal nature of these data. More methodological research is required to fit longitudinal multivariate mixture models in a Bayesian framework.

## F. Potential waning immunity

It is well known that antibodies decay over time, but how much this effects our results is unclear. Unfortunately, we cannot simply compare antibody results from Phase 1 to Phase 2, as these numbers are not directly comparable. Instead, we compared the Phase 1 and Phase 2 probabilities of participants who had a high probability of infection in Phase 1. A comparison of these predicted probabilities is shown in Figure F1. It appears that those with large predicted probabilities in Phase 1 still had large predicted probabilities in Phase 2. This is largely because in Phase 2, we see relatively lower parameter estimates for the means of the infected group. This likely will also make estimates of infection noisier, as the variance will also increase. So although our model does not appear to be underestimating Cumulative Incidence due to waning, waning likely does cause more uncertainty when predicting infection. More work needs to be done to confirm this assertion.
