# Supplementary material for: A Bayesian approach to estimating COVID-19 incidence and infection fatality rates
Source: Biostatistics. 2023 Mar 6;25(2):354–84. doi: 10.1093/biostatistics/kxad003 (PMC11017123; doi:10.1093/biostatistics/kxad003)
Supplement: kxad003_Supplementary_Data [file kxad003_supplementary_data.zip › biosts-22126-File012.pdf]

A Bayesian Approach to Estimating COVID-19 Incidence and Infection Fatality Rates: Reproducible Example

Justin Slater Aiyush Bansal Harlan Campbell Jeffrey S. Rosenthal
Paul Gustafson Patrick E. Brown

2022-10-24

Contents

1 Introduction **1**

2 Data simulation **1**

3 Module 1: Estimating the number of infections in Canada **2**
3.1 Mixture modeling of serosurvey data 2
3.2 Poststratification 5

4 Module 2: Infection Fatality Rates estimation **5**

5 Conclusion **6**

1 Introduction

The serosurvey data used in the main paper is highly confidential and thus cannot be shared publicly. We therefore created this reproducible example using simulated serosurvey data in combination with real census data in order to demonstrate how our method is implemented. We have tried to keep this example as simple as possible, while still retaining the key aspects and novelty of our method. The following simplifications have been made:

1. Instead of using age/sex/ethnicity/education/province as covariates, we are just using age. This lightens the code substantially and will allow us to demonstrate our method without unnecessary details. When poststratifying and computing IFR, we will treat Canada as one region.
2. We are only simulating two titre values instead of three. Since the SmT1 and RBD titre values in Phase 2 tell us generally the same thing, we are going to only simulate NP and SmT1 titre values here. We can use the combination of the two to distinguish between vaccinated and infected individuals.

In this document, we will simulate serosurvey data, with titre values similar to those in our paper. We will replicate the analysis of the Phase 2 data, as this is the most challenging to implement. We present our model setup and stan code for fitting multivariate mixture models to serosurvey titre values, and poststratifying the results. We then demonstrate how we used results from these mixture models, along with disaggregated deaths data in order to estimate Infection Fatality rates in the Canadian population residing outside of long-term care (LTC).

2 Data simulation

We start by simulating serosurvey data where N = 7300, 25% of which are <40 years old, 35% are 40-59 years old, 25% are 60-69 years old, and 15% are 70+ years old. We choose the <40 group to be the reference

Table 1: Incidence overall and by age in simulated serosurvey data

Age	Incidence
Overall	9.67
<40	12.20
40-59	10.10
60-69	8.00
70+	7.20

group, and specify ~12% incidence in this category ($\beta_0 = -1.992$ in the model below). We then make the theoretical incidence in the 40-59 group ~10% ($\beta_1 = -.2$), ~8.4% in the 60-69 year old group ($\beta_2 = -.4$), and ~ 7% in the 70+ group ($\beta_3 = -.6$).

We compute infection probabilities, p_i, for each person $i = 1...N$ in the cohort via:

$$\text{logit}(p_i) = \beta_0 + \beta_1 \cdot \mathbf{1}\{\text{age(i)} \in [40-59]\} + \beta_2 \cdot \mathbf{1}\{\text{age(i)} \in [60-69]\} + \beta_3 \cdot \mathbf{1}\{\text{age(i)} \geq 70]\}$$

where $\mathbf{1}$ is the indicator function. Next, we simulate previous infection status, Z_i, for each person via

$$Z_i = \text{Bern}(p_i)$$

The incidence in our cohort is shown in Table 1. Since this is not a formal simulation study (as this model takes many hours to run), we will take these values as the "true" values of incidence in our simulated serosurvey.

We then assume $\rho = \text{prob}(\text{vaccinated}|Z_i = 0) = 2\%$, and assume nobody was both vaccinated and infected.

We then simulate logged titre values w for each person based on values we found in our paper. We assume shifted and scaled t-distributions, t(df, mean, standard deviation), for each of the titres depending on infection status. We assume $w_{np} \sim t(5, -1.5, 0.1)$ for the uninfected and vaccinated groups, and $w_{np} \sim t(30, -0.5, 0.7)$ for the infected group. Recall that vaccination should have no effect on NP titre values. We assumed $w_{SmT1} \sim t(5, -2, 0.08)$ for the uninfected group, $t(30, -0.5, 1)$ for the infected group, and $t(1, 5, 0.5, 0.05)$ for the vaccinated group. This simulation implies that the NP and SmT1 titre values are independent given infection status.

3 Module 1: Estimating the number of infections in Canada

Estimating the number of infections in Canada is done in two parts. First, we estimate infection probabilities of survey participants based on their age and titre values using a mixture model. Second, since the age distribution of Canada is different than that of the serosurvey, we use poststratification to estimate the number of infections in the Canadian population.

3.1 Mixture modeling of serosurvey data

We now fit our 2-titre, 3-component mixture model to the simulated titre values, assuming we don't know their true infection status. In doing so, we estimate infection probabilities for each survey participant, and use these probabilities to estimate cumulative incidence in Canada via poststratification.

As noted in the paper, we require informative prior information to separate the mixture components, otherwise MCMC is very challenging. In this example, we put a fairly narrow prior centered at 0 for the infected group, intentionally "mis-specifying" this informative prior. Even though this prior has ~99.3% of its mass above the true value, we can recover the true mean titre values in the infected groups of -0.5 reliably (see Table 2). Furthermore, we can see from Table 3 that we can recover the true number of infections in the serosurvey data both overall and by age.

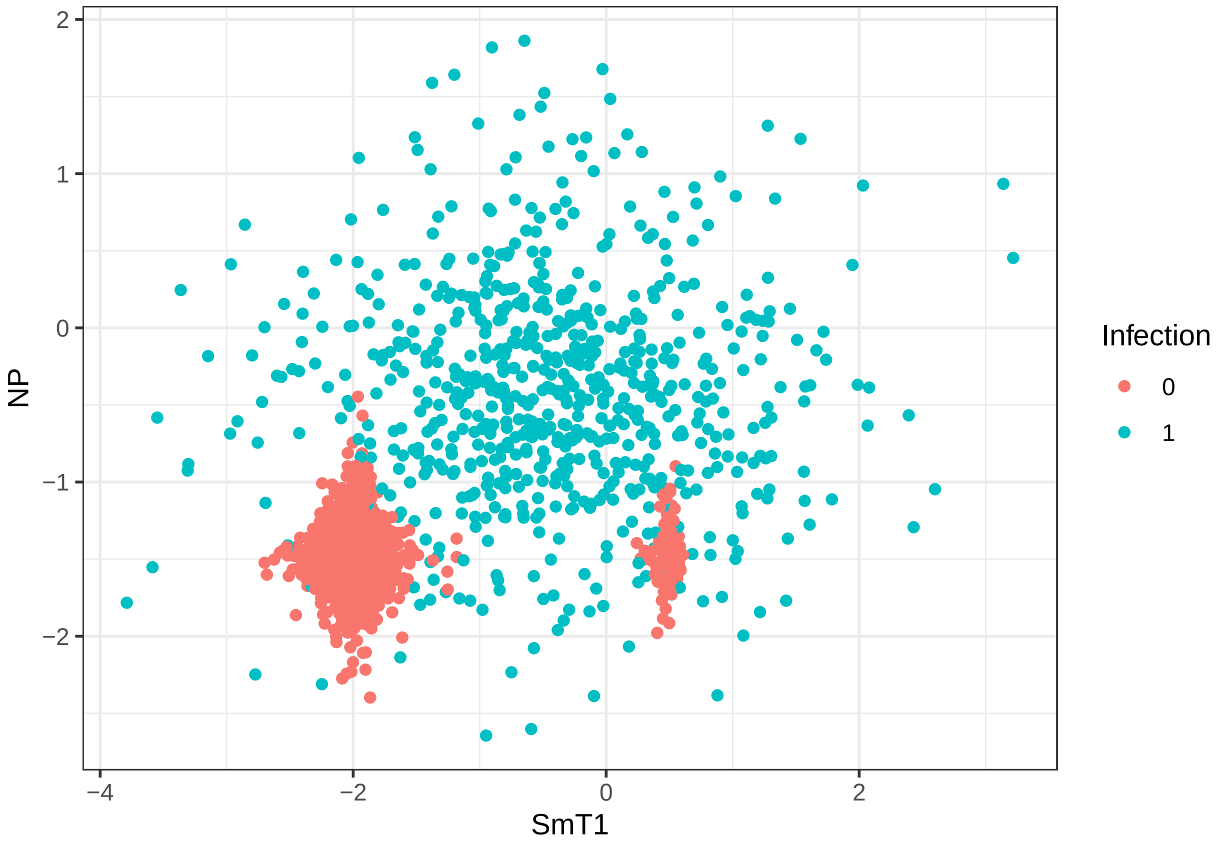

Figure 1: Simulated titre values by infection status. The bottom right cluster of red points corresponds to vaccinated individuals

Table 2: Comparing estimated parameters to true parameters.

	True	Estimate
mu_np[1]	-1.50	-1.5 (-1.503, -1.497)
mu_np[2]	-0.50	-0.44 (-0.499, -0.384)
mu_smt1[1]	-2.00	-1.998 (-2, -1.996)
mu_smt1[2]	-0.50	-0.445 (-0.524, -0.364)
mu_smt1[3]	0.50	0.495 (0.483, 0.506)
rho	0.02	0.018 (0.015, 0.02)

Table 3: Estimated vs. True number of infections in Serosurvey data

Age	Infections	True
Overall	711 (694, 728)	706
<40	220 (213, 227)	222
50-59	258 (249, 268)	259
60-69	150 (144, 155)	146
70+	83 (79, 88)	79

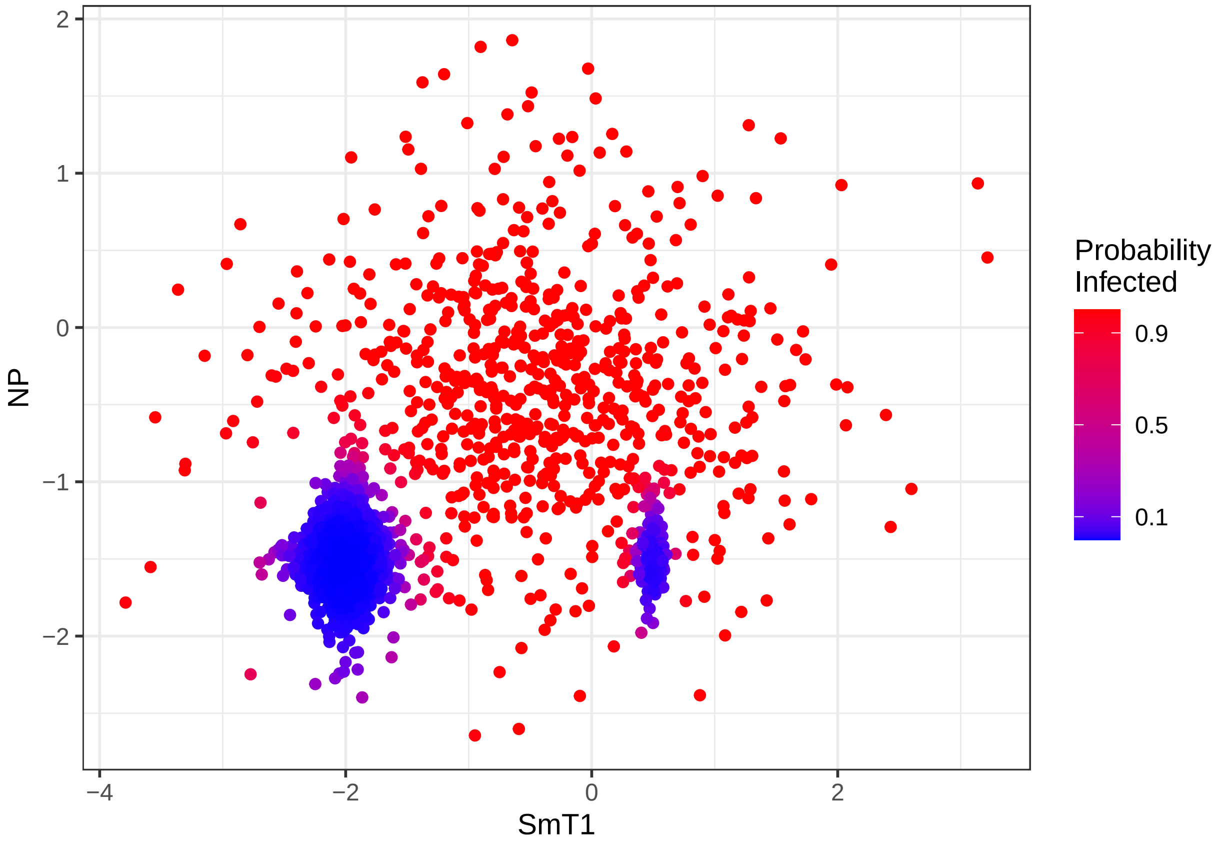

Figure 2: Posterior mean infection probabilities from mixture model for each simulated serosurvey participant.

3.2 Poststratification

The census data has a different age distribution than our simulated serosurvey data, and hence we need to account for this if we want to estimate incidence in Canada.

For each posterior sample, t, of p_j, the number of infections, $Y_j^{(t)}$, in age group j is sampled via

$$Y_j^{(t)} \sim \text{Bin}(n_j, p_j^{(t)})$$

where p_j is the probability that a person in age group j is infected, and n_j is the number of (non-long-term care) people in age category j (this is fixed, and comes from the Census data). Note that in the Canadian population, we don't know a person's titre values (they are "integrated out"), so all we have to predict their infection status is there observed covariates (in this case, age).

As in our paper, the cumulative incidence $I_j^{(t)}$, is then estimated as

$$I_j^{(t)} = \frac{Y_j^{(t)}}{n_j}$$

for each age group j. We can estimate the overall incidence by aggregating posterior samples by group using

$$I_\bullet^{(t)} = \frac{\sum_j Y_j^{(t)}}{\sum_j n_j}$$

The overall incidence, and incidence by age are shown Table 4

4 Module 2: Infection Fatality Rates estimation

In this section, we estimate the infection fatality rates as described in Section 2.4 of the paper. The reason this is challenging is because we only know aggregated numbers of deaths outside of long-term care (LTC), not the deaths by age. This is because our long-term care deaths are not reported by age/sex, and they are a large proportion of the deaths. We do have the total COVID-19 deaths by age, but not the total deaths by age outside of long-term care.

In our paper, we know the deaths by province, but not by age/sex. Here, we know there are 14805 COVID-19 deaths in long-term care, and we have the number of COVID-19 deaths by age, and need to estimate the number of deaths by age outside long-term care, accounting for uncertainty in the age distribution.

We do this by treating the number of infections, estimated from Module 1, as data *for each posterior sample* ($Y_j^{(t)} = y_j^{(t)}$). j is the age category, a subscript of 1 indicates "outside of long-term care", and 2 indicates "within long-term care". For each posterior sample, t, of infections from Module 1, $y_j^{(t)}$, we fit the following model:

$$\begin{aligned}
d_j &\sim \text{Pois}(\lambda_{1j} + \lambda_{2j}) \\
d_2 &\sim \text{Pois}\Big(\sum_j \lambda_{2j}\Big) \\
\lambda_{1j} &= y_j^{(t)} \eta_j \\
\lambda_{2j} &= n_{2j} \theta_j
\end{aligned}$$

where

- d_j is the number of COVID-19 deaths in age group j (LTC + non-LTC)
- d_2 is the number of COVID-19 deaths in long-term care in Canada. 14805 in this case. In the paper, we know this number for each province.
- η_j is the population level infection fatality rate in group j.

Table 4: Incidence and IFR for Canadian population based on simulated serosurvey data. Posterior medians and 95% credible intervals are presented.

Age	Incidence	IFR
Overall	10.2 (9.4, 11)	0.242 (0.221, 0.265)
<40	12.09 (10.57, 13.65)	0 (0, 0.01)
40-59	10.11 (8.98, 11.25)	0.04 (0, 0.08)
60-69	8.18 (6.96, 9.62)	0.19 (0.01, 0.37)
70+	7.62 (6.06, 9.42)	2.46 (1.82, 3.33)

- θ_j is the COVID-19 death rate (Prob infection x IFR). We can't estimate IFR within LTC because we don't know the number of infections in LTC. This is because we don't have serosurvey data from LTC.
- n_{2j} is the long-term care population in age group j.

For each posterior sample from module 1, we obtain 1 posterior sample from this model. We then estimate age-specific IFR outside of long-term care using

$$D_{1j}^{(t)} \sim \text{Bin}(y_{1j}^{(t)}, \eta_j^{(t)})$$

In the paper, we describe how this is equivalent to sampling from a "cut distribution". This accounts for uncertainty in the number of infections as well as the number of deaths when computing IFR, but does not allow deaths data to inform incidence estimates. We present our estimates of IFR for Canada based on our simulated data in Table 4. These numbers are presented for demonstration purposes only and are not reflective of actual incidence or IFR in Canada. Please refer to our paper for our estimates of incidence and IFR.

5 Conclusion

In this short example, we demonstrate how incidence and IFR can be computed using the method described in our paper. We present evidence that our method is not particularly sensitive to the informative priors in our mixture models, and that even if these are mis-specified, we can accurately recover both model parameters as well as incidence estimates. We then show how we estimated IFR using the results of the mixture analysis in combination with disaggregated deaths data.
